# Proteomics Reveals the Role of PLIN2 in Regulating the Secondary Hair Follicle Cycle in Cashmere Goats

**DOI:** 10.3390/ijms26062710

**Published:** 2025-03-18

**Authors:** Cuiling Wu, Qingwei Lu, Shengchao Ma, Nuramina Mamat, Sen Tang, Wenna Liu, Yaqian Wang, Asma Anwar, Yingjie Lu, Qiangqiang Ma, Gulinigaer Aimaier, Xuefeng Fu

**Affiliations:** 1 Xinjiang Key Laboratory of Special Species Conservation and Regulatory Biology, International Center for the Collaborative Management of Cross-Border Pest in Central Asia, College of Life Science, Xinjiang Normal University, Urumqi 830054, China; cuiling_wu@163.com (C.W.); nuramina222@163.com (N.M.); 13579812147@163.com (G.A.); 2 Xinjiang Key Laboratory of Animal Biotechnology, Key Laboratory of Herbivorous Animal Genetics, Breeding and Reproduction, Ministry of Agriculture and Rural Affairs, Institute of Biotechnology, Xinjiang Academy of Animal Sciences, Urumqi 830011, China; lqw120418@163.com (Q.L.); shengchaomasicau@163.com (S.M.); tangsensen610@163.com (S.T.); lwn2362@163.com (W.L.); wangyaqlan@163.com (Y.W.); asma247462@163.com (A.A.); 18199841676@163.com (Y.L.); 15719990471@163.com (Q.M.)

**Keywords:** Jiangnan cashmere goats, secondary hair follicle cycle, proteomics, PLIN2

## Abstract

Based on comprehensive proteomic analysis conducted across various stages of secondary hair follicles (SHFs), the growth and development regulatory mechanisms of SHFs in Jiangnan cashmere goats were studied. Proteomic analysis of skin tissue from the SHF anagen (An), catagen (Cn), and telogen (Tn) revealed 145 differentially expressed proteins (DEPs) between the An and Tn, 53 DEPs between the Cn and An, and 168 DEPs between the Cn and Tn. Gene Ontology (GO) annotations indicated that the DEPs were predominantly involved in keratin filament formation (KRTAP3-1, KRT1, KRT8), intermediate filament formation (KRT26, KRT35, KRT19, etc.), and lipid metabolism (FA2H, CERS6, ECH1, TECR, etc.). Furthermore, Kyoto Encyclopedia of Genes and Genomes (KEGG) pathway enrichment analysis identified significant enrichment of DEPs in pathways related to hair follicle growth and development. Notably, these included the PPAR signaling pathway (PLIN2, PLIN4, ACSL5, etc.), the IL-17 signaling pathway (S100A7A, LOC108633164), and the estrogen signaling pathway (KRT26, KRT35, LOC102176457.). Western blotting (WB) experiments were then performed on five DEPs (KRT28, FA2H, PLIN2, FABP7, and VNN1) to validate the consistency of the WB results with the proteomic data. Overexpression and siRNA interference of *PLIN2* in dermal papilla cells (DPCs) were followed by CCK8 and flow cytometry assays, revealing that *PLIN2* knockdown significantly decreased DPC proliferation while inducing apoptosis, compared to controls. These findings suggest that the *PLIN2* gene plays a crucial role in modulating SHF growth cycles in cashmere goats by influencing DPC proliferation. These results provide novel insights that could inform the development of breeding strategies aimed at enhancing the cashmere yield in such goats.

## 1. Introduction

Jiangnan cashmere goats (*Capra hircus*) represent a newly developed breed that excels in both cashmere and meat production [1]. They are highly valued in the market and among consumers due to the fine, soft texture of their cashmere and its superior thermal insulation properties. However, insufficient cashmere yield remains a significant limitation, constraining the economic benefits of their production. Consequently, optimizing and improving cashmere traits has become a primary objective in breeding programs in Jiangnan cashmere goats.

The hair follicles of cashmere goats are categorized into primary hair follicles (PHFs) and secondary hair follicles (SHFs) [2,3]. PHFs produce coarse hair, which serves to protect against external damage, whereas SHFs generate soft cashmere fibers that play a crucial role in regulating body temperature [4,5,6,7,8]. The growth of SHFs follows a cyclic pattern, encompassing the anagen (An), catagen (Cn), and telogen (Tn) [9,10]. Normally, the secondary hair follicles of Jiangnan cashmere goats are in the An from May to November, the Cn from December to the following February, and the Tn from March to April of the following year. while the changes in the primary hair follicles are not obvious. The An is particularly critical, as it is during this stage that new hair shafts are formed through the proliferation and differentiation of hair matrix cells, which grow upwards to create new fibers. At the core of the hair bulb, the dermal papilla is composed of dermal papilla cells (DPCs), which function as the regulatory center for hair follicle activity. By secreting factors such as matrix metalloproteinases and angiogenesis-related molecules, DPCs promote the proliferation and differentiation of hair matrix cells, thereby regulating follicle growth and angiogenesis. Additionally, DPCs ensure the maintenance of healthy hair follicle growth by modulating the division, proliferation, and differentiation of epidermal cells [11,12,13,14]. The Cn is relatively short, during which the division of hair matrix cells ceases and the lower portion of the follicle regresses. In the Tn, hair growth halts, allowing the follicle to accumulate energy for the subsequent growth cycle. The yield and quality of cashmere are intricately linked to the SHF cycle, a complex biological process involving numerous proteins [15,16,17,18].

Proteins, as the primary executors of cellular functions, represent a tangible manifestation of biological activities. Translated from genetic material, proteins perform a wide range of essential functions in animals, including providing structural support, catalyzing enzymatic reactions, participating in signal transduction, and facilitating material transport. Previous studies demonstrated that certain proteins play crucial roles in hair follicle growth, development, and the hair follicle cycle. For instance, Gao et al. [19], using two-dimensional electrophoresis (2-DE) and mass spectrometry, hypothesized that proteins keratin 1, keratin 5, keratin 25, and keratin 71 may promote hair follicle growth, while keratin 10 and keratin 83 may induce follicles to enter the Tn. Yu et al. [20], employing isobaric tags for relative and absolute quantitation (iTRAQ) protein labeling in conjunction with LC–MS/MS, analyzed skin tissues from Southern Himalayan sheep during the An and Tn, identifying RAC1 as being enriched in signaling pathways affecting the cashmere cycle. Similarly, Yang et al. [21] reported that the upregulation of SFN, CNFN, and KRTAP3-1 contributes to cashmere growth in the skin tissues of Inner Mongolian cashmere goats by using SWATH-based quantitative proteomics. In summary, proteomics research on the hair follicle cycle of cashmere goats provides direct insights into the intrinsic regulatory mechanisms underlying hair follicle cycle-associated phenotypic traits, offering novel approaches for enhancing cashmere yield and quality. However, we still do not know how the protein expression in skin tissues regulates the SHF cycle of Jiangnan cashmere goats.

## 2. Results

### 2.1. Proteomic Characterization Within the Skin Tissues of Cashmere Goats

**Figure 1:**
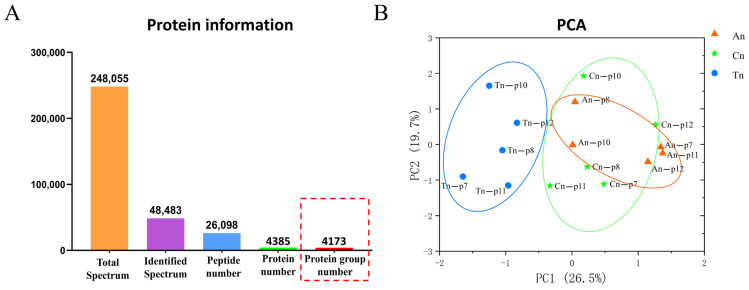
Protein identification in skin tissue of Jiangnan cashmere goats. (**A**). Hierarchical filtering process utilized for protein identification, with distinct colors representing various levels of filtering; (**B**). PCA plot of the proteome, where different colors and shapes signify skin samples from varying stages of secondary follicle development. PC1 and PC2 correspond to the horizontal and vertical axes, respectively.

The comprehensive proteomic characterization of skin tissues across the three SHF developmental stages provided a solid foundation for investigating the molecular mechanisms underlying SHF development. A total of 248,055 secondary mass spectra were obtained from skin tissues across the three SHF developmental stages, with 48,483 spectra successfully mapped to a protein database. From this dataset, 26,098 peptides and 4385 proteins were identified, of which 4173 proteins were functionally annotated for further investigation into their biological roles and mechanisms in SHF development (Figure 1A). Over 50% of the identified proteins possess molecular weights within the range of 10–90 kDa. Furthermore, more than 50% of the proteins demonstrate sequence coverage between 0–40%, suggesting adequate sequence coverage within the proteomics dataset. Approximately 80% of the proteins are composed of fewer than five peptides, with peptide lengths predominantly ranging from 5–21 amino acids (Figure S1A–D). To assess the overall consistency of the proteomics data, principal component analysis (PCA) was performed to cluster the 15 samples. The PCA revealed that the metabolite expression patterns of the samples in the Cn and An groups were similar, while the metabolite expression patterns of the samples in the Tn groups differed significantly from those of the samples in the Cn and An groups (Figure 1B).

### 2.2. Functional Classification of Identified Proteins

By categorizing proteins based on subcellular localization, functional annotations, Gene Ontology (GO) terms, and Kyoto Encyclopedia of Genes and Genomes (KEGG) pathways, we aimed to systematically elucidate their potential involvement in key biological pathways and regulatory networks based on subcellular localization predictions. Proteins in skin tissue exhibited primary distributions as follows: cytoplasm (28.14%), nucleus (27.16%), mitochondria (14.49%), extracellular space (12.76%), plasma membrane (9.44%), cytosol-nucleus (4.21%), endoplasmic reticulum (2.21%), and cytoskeleton (0.65%) (Figure S2A). To further elucidate the functions of the identified proteins, a comprehensive annotation and classification were conducted utilizing functional databases. Among these, 265 proteins were associated with post-translational modification, protein turnover, and chaperones; 264 proteins were broadly associated with functional prediction; 253 proteins were linked to translation and ribosome structure; 145 were potentially involved in signal transduction; 121 were engaged in lipid transport and metabolism; and 102 were associated with energy generation and conversion (Figure S2B).

GO annotation results indicated that, concerning biological processes, proteins were primarily involved in amide biosynthetic processes (GO:0043604), organic acid metabolic processes (GO:0006082), and peptide metabolic processes (GO:0006518). In terms of cellular components, the proteins were predominantly localized to the nucleolus (GO:0005730), intracellular protein-containing complexes (GO:0140535), and supramolecular fibers (GO:0099512). Regarding molecular functions, they were found to participate in cytoskeletal protein binding (GO:0008092), hydrolase activity (GO:0016818), and pyrophosphatase activity (GO:0016462) (Figure S2C).

KEGG pathway annotation revealed that, within cellular processes, the proteins were involved in pathways such as endocytosis (chx04144), regulation of the actin cytoskeleton (chx04810), and focal adhesion (chx04510). In the context of environmental information processing pathways, proteins were associated with the PI3K-Akt signaling pathway (chx04151), the MAPK signaling pathway (chx04010), and the Rap1 signaling pathway (chx04015). For genetic information processing, proteins were linked to the spliceosome (chx03040), the ribosome (chx03010), and the protein processing in the endoplasmic reticulum (chx04141). In pathways associated with human diseases, proteins were predominantly implicated in neurodegenerative diseases (chx05022), amyotrophic lateral sclerosis (chx05014), and Alzheimer’s disease (chx05010). Additionally, within metabolic pathways, proteins were involved in carbon metabolism (chx01200), cofactor biosynthesis (chx01240), and oxidative phosphorylation (chx00190). Furthermore, proteins participated in thermogenesis (chx04714), the complement and coagulation cascades (chx04610), and the NOD-like receptor signaling pathway (chx04621) (Figure S2D).

### 2.3. Differential Expression Analysis of Proteins

**Figure 2:**
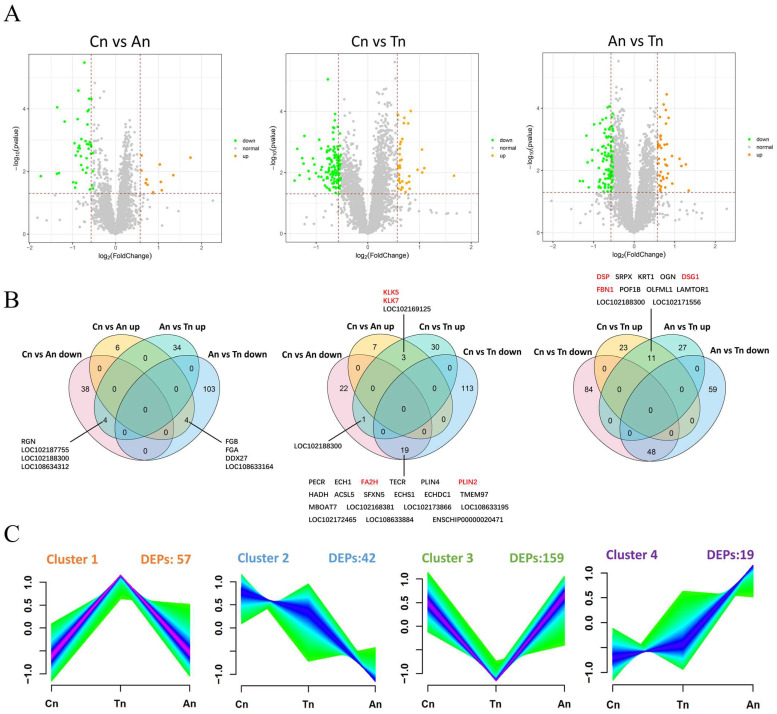
Differential expression analysis of proteins. (**A**–**C**) illustrate the volcano plot, Venn diagrams, and expression trend maps of differentially expressed proteins across various comparison groups. The figure presents Venn diagrams illustrating the up and down regulated differential proteins across various comparison groups. Proteins associated with hair follicle growth and development are indicated in red font. The color gradient from green to blue indicates the density of protein expression patterns across different stages (Cn, Tn, An) in the trend maps.

To elucidate the molecular mechanisms underlying hair follicle development in Jiangnan cashmere goats, this study used tandem mass tag (TMT) technology to construct protein expression profiles at different developmental stages in the skin of five Jiangnan cashmere goats. The expression profiles were compared across three distinct groups: (1) Cn vs. An; (2) Cn vs. Tn; (3) An vs. Tn. The analysis of Cn vs. An revealed 53 differentially expressed proteins (DEPs), which included 11 upregulated and 42 downregulated DEPs. In Cn vs. Tn, 168 DEPs were identified, consisting of 35 upregulated and 133 downregulated DEPs. In An vs. Tn, a total of 145 DEPs were identified, comprising 38 upregulated and 107 downregulated DEPs (Figure 2A).

Figure 2B further elucidates the overlap of upregulated and downregulated DEPs associated with hair follicle growth and development. A total of 189 DEPs were identified at the intersection of Cn vs. An and An vs. Tn. Among these, four proteins (RGN, LOC102187755, LOC102183300, and LOC108634312) were downregulated in the Cn vs. An group but upregulated in the An vs. Tn group, while another four proteins (FGB, FGA, DDX27, and LOC108633164) were upregulated in the Cn vs. An group but downregulated in the An vs. Tn group. In the intersection of Cn vs. An and Cn vs. Tn, a total of 195 DEPs were identified. Among these, three proteins (KLK5, KLK7, and LOC102169125) were found to be upregulated, while 19 proteins (FA2H, PLIN2, etc.) were observed to be downregulated. In the intersection of Cn vs. Tn and An vs. Tn, a total of 252 DEPs were identified, with 11 proteins (DSP, DSG1, FBN1, etc.) exhibiting upregulation.

Cluster analysis of expression patterns delineated four unique clusters (Figure 2C and Figure S3). Cluster 1 contained 57 DEPs exhibiting consistently increased expression in Cn and Tn; Cluster 2 consisted of 42 DEPs demonstrating a steady decrease in expression from Cn to An; Cluster 3 comprised 159 DEPs that showed decreased expression in the Cn and Tn but increased expression between Tn and An; and Cluster 4 included 19 DEPs with a continuous increase in expression from Cn to An. Subsequent clustering analysis of DEPs across the three groups revealed a distinct differential protein expression pattern during An in comparison to Cn and Tn (Figure S4).

### 2.4. Functional Enrichment Analysis and PPI (Protein–Protein Interaction) Network Analysis of DEPs

**Figure 3:**
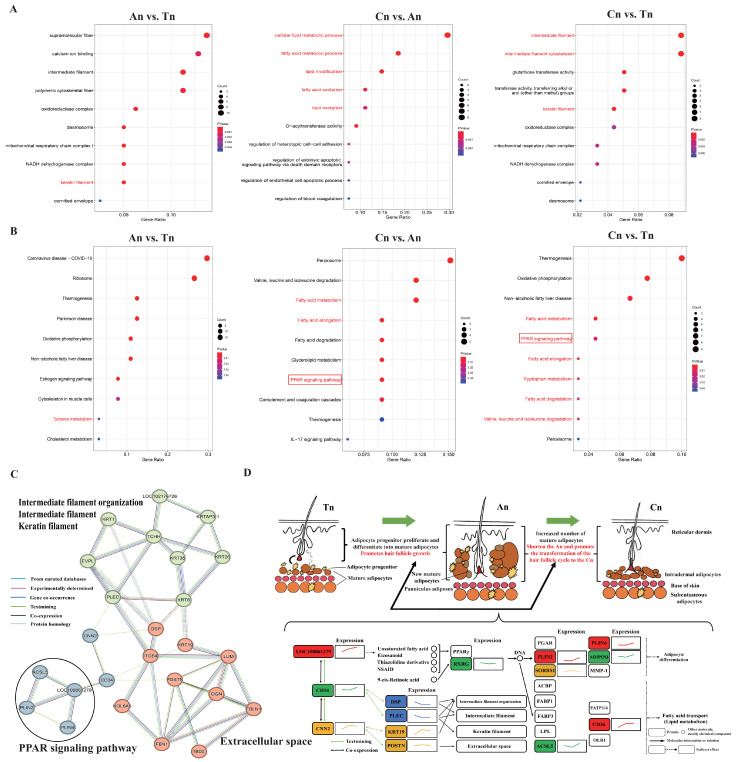
The potential roles of DEPs in regulating the hair follicle cycle. (**A**), GO analysis of DEPs across the three developmental stages. (**B**), KEGG analysis of DEPs across the three developmental stages. (**A**,**B**) figure: Red Font: Biological processes and pathways potentially associated with the hair follicle cycle. Red Box: Key pathways for further analysis. (**C**), PPI network analysis of selected DEPs. This network underscores the potential interactions among DEPs implicated in the PPAR signaling pathway, intermediate filament organization, and keratin filament dynamics. (**D**), Schematic representation delineating the effects of adipocyte differentiation and growth on the hair follicle cycle and the PPAR signaling pathway. The diagram illustrates the interactions between DEPs within the PPAR signaling pathway and those involved in other pathways, as well as the expression trends of DEPs during the transitions from Tn to An to Cn. The colors assigned to the DEPs correspond to the colors of their respective expression trend lines.

Elucidating the biological functions and regulatory networks of DEPs in hair follicle development, we performed functional enrichment and PPI network analyses to uncover key pathways and molecular interactions driving follicle growth across stages. In An vs. Tn, DEPs were primarily enriched in terms or pathways related to cytoskeleton and structural organization, including intermediate filament (GO:0005882), keratin filament (GO:0045095), polymeric cytoskeletal fiber (GO:0099513), and ribosome (chx03010) (Figure 3A,B). In Cn vs. An, DEPs were significantly enriched in terms or pathways associated with lipid metabolism, adipocyte growth, and differentiation, such as cellular lipid metabolic process (GO:0044255), fatty acid metabolic process (GO:0006631), lipid oxidation (GO:0034440), fatty acid metabolism (chx01212), fatty acid elongation (chx00062), glycerolipid metabolism (chx00561), and PPAR signaling pathway (chx03320) (Figure 3A,B). In Cn vs. Tn, DEPs were enriched in terms or pathways also related to hair structure, lipid metabolism, and adipocyte growth and differentiation, including intermediate filament (GO:0005882), keratin filament (GO:0045095), intermediate filament cytoskeleton (GO:0045111), PPAR signaling pathway (chx03320), fatty acid elongation (chx00062), and fatty acid degradation (chx00071). Additionally, some other DEPs were also enriched in oxidoreductase complex (GO:1990204) and oxidative phosphorylation (chx00190) (Figure 3A,B).

The DEPs identified in the Cn vs. An, Cn vs. Tn, and An vs. Tn were then used for PPI network analysis (Figure 3C). The findings indicated that KRT8, KRT19, and KRT35 are situated at pivotal nodes within the intermediate filament organization, intermediate filament, and keratin filament pathways. Furthermore, these pathways are interconnected with the extracellular space through intermediate nodes such as DSP, ITGB4, and KRT19, thereby underscoring a collaborative relationship between the cytoskeleton and the extracellular matrix. The PPAR signaling pathway appears to function independently of other terms or pathways. However, it retains certain interactions with other terms or pathways through intermediate nodes such as CNN2 and CD. It is worth noting that LOC100861279 is positioned upstream in the PPAR pathway, demonstrating an upregulation expression trend from the An to Tn. PLIN2, PLIN4, and CD36 are located downstream, all of which exhibited an upregulated expression trend from the An to Tn. RXRG and ADIPOQ (or ACSL5) are located midstream and downstream, respectively, all of which showed a downregulated and then upregulated expression trend from the An to Tn (Figure 3D).

### 2.5. Protein Western Blot Analysis

**Figure 4:**
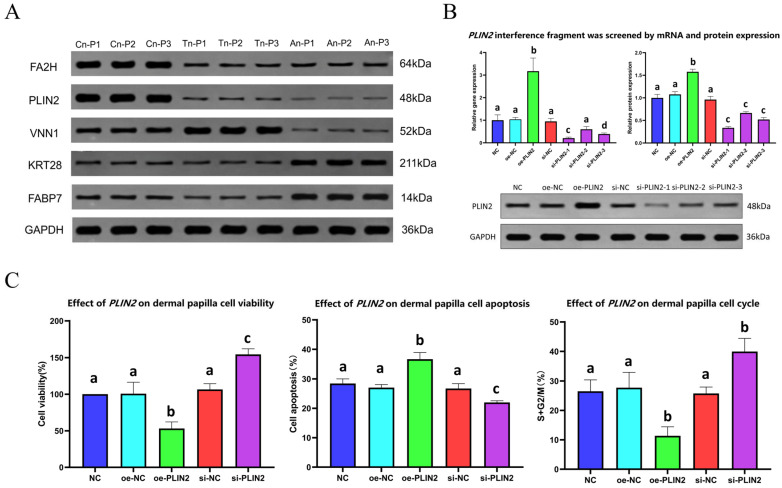
Western blot analysis, *PLIN2* gene interference screening, and impact of *PLIN2* on DPCs viability, apoptosis, and growth cycle regulation in Jiangnan cashmere goats. (**A**), Western blot was performed to assess the expression of FA2H, PLIN2, VNN1, KRT28, and FABP7, with GAPDH serving as a reference protein. Cn-P1, Cn-P2, and Cn-P3 correspond to Cn skin samples, Tn-P1, Tn-P2, and Tn-P3 correspond to Tn skin samples, and An-P1, An-P2, and An-P3 correspond to An skin samples. (**B**), Screening of *PLIN2* interference fragments via mRNA and protein levels. (**C**), Effects of *PLIN2* gene on DPC viability, apoptosis, and cell cycle. Data are presented as mean ± SEM. Statistical analysis of experimental results was conducted using the *t*-test; different lowercase letters indicate significant differences (*p* < 0.05), while identical lowercase letters denote insignificant differences (*p* > 0.05).

Five proteins (FA2H, PLIN2, VNN1, KRT28, and FABP7) were selected for protein Western blot analysis to detect the expression levels of these proteins in the skin during different SHF development phases. The results demonstrated that the trend in the abundance of the detected proteins was consistent with the findings from the proteomics analysis, thereby further substantiating the reliability and accuracy of the proteomics data (Figure 4A and Figure S5).

### 2.6. Screening of PLIN2 Gene Interference Fragments

The most effective *PLIN2* gene interference fragment was identified by transfecting three synthesized sequences into DPCs and evaluating their interference efficiency through mRNA and protein expression analysis, following the transfection of three synthesized *PLIN2* gene interference fragments into DPCs for a duration of 48 h; the mRNA expression levels of the *PLIN2* gene were evaluated using qRT-PCR (Figure 4B). The results indicated that relative to the negative control (NC) group, the *PLIN2* expression level in the oe-NC group was elevated, although this increase was not statistically significant. In contrast, the oe-PLIN2 group exhibited a significant increase in *PLIN2* expression (*p* < 0.05). The si-NC group demonstrated a reduction in *PLIN2* expression; however, this decrease was not statistically significant. The si-PLIN2-1 group exhibited a significant reduction in *PLIN2* expression (*p* < 0.05), while si-PLIN2-2 showed a decrease that lacked statistical significance. Additionally, the si-PLIN2-3 group also presented a significant reduction in *PLIN2* expression. Accordingly, si-PLIN2-1 exhibited the most pronounced interference effect, followed by si-PLIN2-3, with si-PLIN2-2 demonstrating the weakest effect. The oe-NC group then exhibited an increased *PLIN2* expression level relative to the NC group, although this difference was not statistically significant. The oe-PLIN2 group displayed a significant enhancement in *PLIN2* expression (*p* < 0.05). The si-NC group revealed a decrease in *PLIN2* expression, which was not statistically significant (*p* > 0.05). In comparison, the *PLIN2* expression levels in the si-PLIN2-1, si-PLIN2-2, and si-PLIN2-3 groups were all significantly reduced (*p* < 0.05), with si-PLIN2-1 exhibiting the lowest protein expression level (Figure 4B). Western blot analysis further corroborated that si-PLIN2-1 had the lowest grayscale value, indicating the minimal protein expression level (Figure 4B). Consequently, si-PLIN2-1 was selected as the *PLIN2* gene interference fragment for subsequent experiments.

### 2.7. Effects of the PLIN2 Gene on DPC Proliferation, Apoptosis, and Growth Cycle

To explore the functional role of *PLIN2* in DPCs, its effects on cell proliferation, apoptosis, and cell cycle progression were assessed. *PLIN2* overexpression and knockdown were employed to investigate its regulatory influence on DPC growth dynamics. Following the transfection with the *PLIN2* gene overexpression vector and si-PLIN2-1 control, cell proliferation rates were evaluated using the cell counting kit-8 (CCK-8) assay (Figure 4C). The proliferation rates were as follows: NC group, 100.000%; oe-NC group, 100.646 ± 15.668%; oe-PLIN2 group, 53.151 ± 9.001%; si-NC group, 106.558 ± 7.834%; and si-PLIN2 group, 154.240 ± 7.514%. Compared to the NC group, the oe-NC group exhibited a slight increase in proliferation rate, although this was not statistically significant (*p* > 0.05). Conversely, the oe-PLIN2 group demonstrated a significant decrease in cell proliferation rate (*p* < 0.05). While the si-NC group displayed an increase in proliferation rate without significant difference (*p* > 0.05), the si-PLIN2 group showed a significant increase (*p* < 0.05). These findings indicate that interference with the *PLIN2* gene effectively promotes cell proliferation.

The apoptosis of DPCs was assessed by flow cytometry following transfection with the *PLIN2* overexpression vector and si-PLIN2. The apoptosis rates were as follows: NC group, 28.445 ± 1.553%; oe-NC group, 27.038 ± 1.033%; oe-PLIN2 group, 36.681 ± 2.187%; si-NC group, 26.713 ± 1.650%; and si-PLIN2 group, 21.98 ± 0.565%. Compared to the NC group, the apoptosis rate in the oe-NC group showed a slight decrease, although this was not statistically significant (*p* > 0.05). In contrast, the oe-PLIN2 group exhibited a significant increase in apoptosis rate (*p* < 0.05). The si-NC group demonstrated a reduction in apoptosis rate without a significant difference (*p* > 0.05), whereas the si-PLIN2 group exhibited a significant reduction in apoptosis (*p* < 0.05) (Figure 4C). These results suggest that interference with the *PLIN2* gene can inhibit cell apoptosis.

Flow cytometry was also employed to analyze the cell cycle distribution of DPCs (Figure 4C). In comparison to the NC group, the S+G2/M ratio in the oe-NC group increased but was not statistically significant (*p* > 0.05). However, the S+G2/M ratio in the oe-PLIN2 group showed a significant decrease (*p* < 0.05). The si-NC group exhibited a decrease in S+G2/M ratio without significance (*p* > 0.05), while the si-PLIN2 group demonstrated a significant increase (*p* < 0.05). Flow cytometry analysis revealed that disruptions in *PLIN2* expression significantly increased the proportion of cells in the S and G2/M phases, suggesting an acceleration of cell cycle progression.

## 3. Discussion

Hair follicles are unique skin appendages capable of cyclical growth, a characteristic that imparts specific biological traits to cashmere goats, significantly influencing cashmere yield and fiber quality [22,23]. The growth and development of hair follicles are regulated by various genes and signaling pathways, with numerous genes and proteins identified as being associated with follicular development in cashmere goats [24,25]. However, there has been limited research on the proteomic changes and molecular mechanisms underlying the secondary hair follicles of Jiangnan cashmere goats Therefore, this study aimed to fill the knowledge gap in cashmere growth and provide a theoretical foundation for understanding its underlying regulatory mechanisms. In this study, three groups (Cn vs. An; Cn vs. Tn; An vs. Tn) were established to investigate DEPs during different SHF phases. We finally identified 145, 53, and 168 DEPs in the three groups, respectively. These findings highlight significant differences in protein expression at various developmental stages, indicating that complex molecular regulatory mechanisms are involved in SHF development. These mechanisms are likely closely associated with the distinct stages of the hair follicle growth cycle. In the following sections, the potential regulatory mechanisms of these DEPs on hair follicle cycle are discussed.

### 3.1. The Effect of Differentially Expressed Calcium-Binding Proteins on the SHF Cycle

In the present study, S100A7A exhibited elevated expression levels during the An, with a significant downregulated pattern observed in the An vs. Cn and Cn vs. Tn. S100A7A, a member of the S100 calcium-binding protein family, is primarily involved in regulating cell migration, invasion, differentiation, and cell cycle progression [26]. Research has also indicated that S100A7A is upregulated within the epidermal differentiation complex (EDC) gene family, which, either individually or cooperatively, promotes keratinocyte differentiation, thereby influencing the differentiation of both the epidermis and hair follicles [27]. The upregulation of S100A7A may enhance its immunoregulatory function and be associated with inflammatory responses and keratinocyte differentiation, a phenomenon particularly pronounced in proliferative skin disorders such as psoriasis [28]. Therefore, we hypothesize that the diminished expression of S100A7A contributes to impaired hair follicle development, subsequently facilitating the transition of the hair follicle cycle from An to Tn.

KEGG enrichment analysis further showed that the S100A7A was significantly enriched in the IL-17 signaling pathway. This pathway influences hair follicle growth and cycling by modulating inflammation and cell proliferation. Studies have demonstrated the pivotal role of IL-17 signaling in regulating various phases of the hair follicle cycle. For instance, Wu et al. [29] conducted transcriptomic analyses of secondary hair follicles in Jiangnan cashmere goats and identified significant differences in the expression of IL-17-related genes across developmental stages, thereby underscoring the pathway’s regulatory importance in secondary hair follicle development. Furthermore, Zhang et al. [30], utilizing 2D and 3D dermal papilla cell models, investigated the effects of dihydrotestosterone (DHT) on hair follicle growth. Their findings revealed that the IL-17 pathway is not only involved in inflammatory responses but may also be closely linked to androgenetic alopecia. This further supports that S100A7A regulates the hair follicle cycle by participating in the IL-17 signaling pathway.

### 3.2. The Effect of Differentially Expressed Keratins on the SHF Cycle

Keratins not only determine the quality of wool and cashmere but are also closely linked to hair follicle growth [31,32]. In this study, KRT26 and KRT35 were downregulated in the An vs. Tn and were significantly enriched in the estrogen signaling pathway. Both KRT26 and KRT35 belong to the keratin (KRT) family, whose proteins constitute the main structural components of wool fibers. These proteins primarily provide mechanical stability and elasticity to the fibers by forming keratin intermediate filaments within the fiber matrix [33,34].

KRT35 is a key protein that regulates hair growth and fineness and plays a central role in hair follicle development and fiber formation [35]. According to Langbein et al. [35], both KRT35 and KRT85 belong to the family of type I keratins, and KRT35 is not only involved in the structural formation of hair, but also has a close relationship with the hair growth cycle and keratinization process. In addition, the co-expression pattern of KRT35 with other keratin proteins (e.g., KRT38) helps to define the morphological characteristics of hairs, especially hair curvature [36]. Additionally, its presence in only one specific cell type within the mature fiber suggests a potential role in defining fiber curvature [37]. Jin et al. [38] conducted RNA interference experiments on the *KRT26* gene of Liaoning cashmere goats to investigate its effects on keratinocyte proliferation and differentiation. The results revealed that KRT26 inhibits cell proliferation and differentiation and is closely associated with the transition of hair follicles between the Cn and Tn phases. Duchstein et al. indicated that KRT26 plays a significant role in hair follicle morphogenesis and skin development, thereby regulating cashmere fiber fineness [39]. Hui et al. [40] investigated the skin transcriptomes of two breeds of cashmere goats and found that KRT26 and KRT35 may cooperatively regulate cashmere growth and fineness. In summary, the findings of Jin et al. [38] appear to be contrary to our results but are consistent with those of Duchstein et al. [39] and Hui et al. [40] The findings of Jin et al. appear to contradict our results but align with those of Duchstein et al. [39] and Hui et al. [40] Several factors may account for these discrepancies. First, genetic differences among breeds could influence KRT26 expression and function, thereby affecting hair follicle development and fiber characteristics. While Jin et al. studied Liaoning cashmere goats, our research focused on Jiangnan cashmere goats, which may have led to variations in results. Second, methodological differences may have contributed to the contrasting findings. Jin et al. employed RNA interference (RNAi) in keratinocytes to investigate the role of KRT26, whereas we utilized proteomic approaches to examine its expression across different stages of the hair follicle cycle. These differences in experimental design could have influenced the outcomes. Third, environmental and physiological factors—such as seasonal changes, nutrition, and hormonal fluctuations—may have impacted KRT26 expression. Variations in study conditions could explain the observed differences in hair follicle regulation. Despite these inconsistencies, our findings are consistent with those of Duchstein et al. [39] and Hui et al. [40], further supporting the crucial role of KRT26 in hair follicle development and fiber formation. Our study supports the conclusion that KRT26 and KRT35 promote hair follicle growth and development. Estrogen plays a critical regulatory role in skin physiology and hair growth. Studies have shown that the estrogen signaling pathway, mediated through estrogen receptors α (ERα) and β (ERβ), significantly affects the Cn and Tn phases of the hair follicle cycle. The topical application of 17β-estradiol (E2) can arrest hair follicles in the Tn phase, thereby inhibiting hair growth [41]. Furthermore, unliganded estrogen receptors can induce the transition of hair follicles into the Cn [42,43]. These findings suggest that KRT26 and KRT35 play roles in hair follicle development and cyclical changes through the estrogen signaling pathway.

### 3.3. The Effect of Differentially Expressed Ribosomal Proteins on the SHF Cycle

RPS5, RPL26, and RPL32 are members of the ribosomal protein family. Ribosomal proteins (RPs) are integral components of the ribosomal structure and include both large and small subunit proteins [44]. Zhang et al. [44], through multi-omics analyses, demonstrated that ribosomal proteins may regulate differentiation, development, and apoptosis during various stages of hair follicle development in Inner Mongolia cashmere goats.

In this study, ribosomal proteins were significantly enriched in pathways related to translation, peptide biosynthesis, and amide synthesis in the Cn vs. Tn and An vs. Tn groups (RPS2, RPS9, RPS18, RPS24, RPS26 etc.). Additionally, the expression levels of RPL family proteins were significantly upregulated during the Cn and An phases (RPL11, RPL13, RPL14, RPL15, etc.). These results suggest that ribosomal proteins may regulate hair follicle cells through multiple biological pathways, influencing the growth, regression, and regeneration of SHFs in Jiangnan cashmere goats.

### 3.4. DEPs Linked with Lipid Metabolism Affect SHF Cycle

Previous studies demonstrated that an increased number of adipocyte precursor cells with proliferative and differentiative potential in dermal adipose tissue accelerate the transition of hair follicles into the An phase [45]. During the mid-to-late stages of An, the number of mature adipocytes and the thickness of the dermal adipose tissue layer reach their peak [45]. Mature adipocytes contribute to the shortening of the An phase and promote the transition of the hair cycle into the Cn phase [46]. The proliferation and differentiation of adipocytes are intrinsically linked to intracellular lipid synthesis and are regulated by complex molecular mechanisms. Meanwhile, our study revealed that four DEPs were enriched in the fatty acid metabolism pathway in Cn vs. An and Cn vs. Tn groups. Therefore, we hypothesize that these DEPs may influence the hair follicle cycle by participating in lipid metabolic pathways. Notably, LOC100861279, PLIN2, and ACSL5 were found to be enriched in the PPAR signaling pathway. Previous research established the critical role of the PPAR signaling pathway in skin and hair follicle development, particularly concerning fibroblast and epithelial cell development, hair follicle morphogenesis, and skin development [47,48]. Zhou et al. [49] observed significant enrichment of the PPAR signaling pathway across various stages of the hair follicle cycle in Tianzhu white yaks. A key function of PPAR involves its participation in lipid metabolism and its influence on adipocyte proliferation and differentiation. Consequently, the regulation of the hair follicle cycle by the PPAR signaling pathway may occur via its effects on adipocyte proliferation and differentiation.

From Tn to An and subsequently from An to Cn, LOC100861279, PLIN2, and PLIN4 exhibited an upregulated expression trend. These proteins are situated both upstream and downstream within the PPAR signaling pathway, indicating that the adipocyte differentiation-related branch of this pathway is continuously enhanced during these transitions. Based on this observation, we speculate that the sustained enhancement of this pathway from Tn to An promotes accelerated hair follicle development. However, once a peak is reached, further enhancement of the pathway may decelerate hair follicle development, leading to the transition from An to Cn in the SHF developmental stage. Yin et al. discovered that the PPAR signaling pathway influences hair follicle growth and cyclical transitions in androgenetic alopecia (AGA) by regulating adipocyte function and lipid metabolism [50]. Their findings align with and support the conclusions of this study.

In mature adipocytes, lipid droplets function as cellular structures for the storage of neutral lipids, which are primarily composed of triacylglycerols (TAGs) and cholesteryl esters (CEs), surrounded by a phospholipid monolayer. PLIN2 (Perilipin 2), a protein essential for lipid metabolism, is located on the surface of lipid droplets and is primarily involved in lipid metabolism and cellular signaling [51]. *PLIN2* may regulate lipid droplet formation, storage, and release via the PPAR signaling pathway, thereby influencing energy metabolism and the cyclical changes of secondary hair follicles. However, despite its recognized importance in lipid metabolism, the role of *PLIN2* in hair follicle cells has not been thoroughly investigated, and its molecular mechanism in regulating hair follicle growth and development remains largely unexplored. To address this issue, the study designed and synthesized vectors for *PLIN2* overexpression and interference fragments. Using transient transfection methods, the effects of these fragments on DPCs of secondary hair follicles in cashmere goats were examined to elucidate the role of *PLIN2* in the hair follicle cycle. The results demonstrated that *PLIN2* interference significantly promoted DPCs proliferation, whereas its overexpression inhibited DPC proliferation. Previous studies showed that lipids stored in lipid droplets can be degraded via autophagy. Notably, *PLIN2* overexpression inhibits autophagy, leading to the continuous accumulation of lipid droplets within cells [52,53,54]. Based on these findings, we hypothesize that *PLIN2* overexpression increases lipid accumulation within DPCs, thereby suppressing their proliferation. Conversely, reduced *PLIN2* expression promotes autophagy, decreases lipid droplet accumulation, and enhances DPC proliferation, ultimately supporting hair growth. These results provide strong evidence that the *PLIN2* gene plays a critical regulatory role in the secondary hair follicle cycle of cashmere goats, offering new insights into the molecular mechanisms underlying hair follicle development.

## 4. Materials and Methods

### 4.1. Experimental Animals and Sample Collection

Five female Jiangnan cashmere goats, each 24 months of age, were selected for this study. These goats were raised in a shared environment with unrestricted access to food and water. Skin samples were collected during three distinct phases of the secondary hair follicle cycle: anagen (An, n = 5) in September, catagen (Cn, n = 5) in January, and telogen (Tn, n = 5) in March. After depilation of the scapular hair and administration of local anesthesia to each goat, a 1 cm^2^ skin sample was taken using a 10 mm diameter skin sampler. Yunnan Baiyao hemostatic powder (Yunnan Baiyao company, Kunming, China) was uniformly applied to the skin wound of the goats. The samples were subsequently rinsed with phosphate-buffered saline (PBS), divided into cryotubes, and stored at −80 °C, resulting in a total of 15 samples. All animal experiments were approved by the Biology Ethics Committee of the Xinjiang Academy of Animal Sciences.

### 4.2. Protein Extraction and Digestion

This study examined the quantification of proteins in skin tissues through the utilization of the TMT labeling technique. Initially, the skin samples were finely ground into a powdered form. Subsequently, the proteins were extracted through centrifugation after the addition of urea lysis buffer and protease inhibitors. A 50 µg protein aliquot from each sample underwent reduction, followed by the addition of dithiothreitol (Amresco, Philadelphia, PA, USA) and ammonium bicarbonate (Sigma-Aldrich, St. Louis, MO, USA) dilution buffer. The proteins were then digested with trypsin (Promega, Madison, WI, USA) and incubated overnight at a temperature of 37 °C. After this step, iodoacetamide (Amresco, Philadelphia, PA, USA) was introduced for alkylation, and the pH was adjusted. Subsequently, trypsin (Promega, Madison, WI, USA) was added once again to facilitate further digestion. Finally, the samples were processed utilizing C18 desalting columns (Waters Corporation, Milford, CT, USA) and subsequently lyophilized for storage purposes.

### 4.3. TMT Labeling

After thawing the TMT labeling reagent (Thermofisher, Waltham, MA, USA) at room temperature, 41 µL of acetonitrile (Avantor, Radnor, PA, USA) was added. The mixture was vigorously mixed for 5 min, followed by centrifugation. Subsequently, the TMT reagent was added to 100 µg of digested protein sample and incubated at room temperature for 1 h. The reaction was terminated by adding ammonia solution (Fujifilm Wako Chemicals, Osaka, Japan). The labeled samples were combined, thoroughly mixed using a vortex mixer (SCILOGEX, Rocky Hill, CT, USA, Model: MX-S), and centrifuged to collect the solution at the bottom of the tube. Finally, the samples were subjected to desalting and lyophilization to concentrate the peptides for subsequent mass spectrometry analysis. This experimental protocol enables accurate quantification of proteins in skin tissues, thereby providing essential data support for further biological research and diverse applications.

### 4.4. LC–MS/MS Analysis

Liquid chromatography–tandem mass spectrometry (LC–MS/MS) was performed using the RIGOL L-3000 system(RIGOL Technologies, Inc., Beijing, China), which utilized mobile phase A (100% water, 0.1% formic acid) and mobile phase B (80% acetonitrile, 0.1% formic acid). The lyophilized samples were reconstituted in mobile phase A, centrifuged, and 1 µg of the supernatant was injected for LC–MS analysis. A Q Exactive HF-X mass spectrometer equipped with a Nanospray Flex™ (Thermofisher, Waltham, MA, USA) ion source was employed, with a spray voltage of 2.4 kV and an ion transfer tube temperature of 275 °C. Data-dependent acquisition was utilized, with a scan range of *m*/*z* 407–1500, a resolution of 60,000, and an automatic gain control target of 3×. The top 40 precursor ions were selected for higher-energy collisional dissociation (HCD), operating at a resolution of 45,000 and an automatic gain control target of 5 × 10^4^, thereby generating raw spectral data.

### 4.5. Protein Characterisation and Quantitation

The mass spectrometry data generated in this study were analyzed using Proteome Discoverer software (version 2.4). The search parameters included trypsin digestion with a maximum of two allowed missed cleavages. Carbamidomethylation of cysteine (C) was considered as a fixed modification, while methionine oxidation (+15.995 Da) and TMT-6plex labeling (K- and N-terminal) were treated as dynamic modifications. The precursor ion mass tolerance was set to ±10 ppm, and the fragment ion mass tolerance was set to ±0.02 Da. Protein accession numbers were converted to gene symbols utilizing the UniProt Retrieve/ID Mapping tool (https://www.uniprot.org/id-mapping, accessed on 17 February 2024), and functional annotation was conducted using the UniProt database (http://www.ebi.ac.uk/GOA/, accessed on 24 February 2024). The subcellular localization of proteins was predicted with Wolfpsort (http://www.genscript.com/psort/wolf_psort.html, accessed on 5 March 2024) [55].

### 4.6. Bioinformatic Analysis

Differential expression analysis was performed using edgeR (version 4.2.1) [56], with proteins meeting the criteria of fold change > 1.5 or <0.7 and *p* < 0.05 designated as upregulated or downregulated DEPs. KEGG pathway enrichment analysis was conducted using the KEGG database (https://www.kegg.jp/kegg, accessed on 12 March 2024) [57], while Gene Ontology (GO) annotation included biological processes, cellular components, and molecular function GO terms and KEGG pathways with *p* < 0.05 were regarded as significantly enriched. Key genes were identified, and protein–protein interaction networks were constructed using the STRING 12.0 database [58], with a confidence threshold set at ≥0.4.

### 4.7. Western Blot Analysis of DEPs

Proteins were extracted from the remaining skin samples for Western blotting (WB) analysis of candidate DEPs to analyze the expression patterns of these candidate proteins: KRT28 (anti-mouse antibody, JC838713S), FA2H (anti-rabbit antibody, bs-5104R), PLIN2 (anti-rabbit antibody, bs-1164R), FABP7 (anti-rabbit antibody, PK27766S), and VNN1 (anti-rabbit antibody, bs-10314R). The procedure involved protein separation, electrophoresis, membrane transfer, blocking, and incubation with primary and secondary antibodies, followed by ECL chemiluminescent detection. The anti-mouse secondary antibody was diluted at 1:5000, and the anti-rabbit secondary antibody at 1:3000 The WB analysis of these candidate proteins further validated the results of the proteomic sequencing.

### 4.8. Functional Validation of the PLIN2 Gene in DPCs

Based on the proteomic analysis of skin tissue from various stages of the SHF cycle in Jiangnan cashmere goats, the *PLIN2* gene was identified as a potential regulator of SHF cycle development. Functional validation of *PLIN2* was conducted in DPCs using the quantitative reverse transcription–polymerase chain reaction (qRT-PCR), Western blot, and CCK-8 assays to assess its effect on DPCs growth.

#### 4.8.1. Synthesis of PLIN2 Gene Interference Fragments

Three interference sequences targeting the *PLIN2* gene were designed and synthesized by Gene Pharma (Shanghai, China) with the following sequences shown in Table 1.

#### 4.8.2. Construction of PLIN2 Gene Overexpression Vector

The coding sequence (CDS) of the goat *PLIN2* gene (XM_018051766.1) was retrieved from the NCBI database, and the target fragment was synthesized. This fragment was subsequently ligated with the pcDNA3.1(+) vector (Genepharma, Suzhou, China) at 4 °C overnight. Following the ligation reaction, 5 µL of the ligated product was introduced into competent cells, which were then supplemented with LB medium for 1 h recovery culture at 37 °C with shaking at 200 rpm. Upon recovery, the bacterial suspension was streaked onto LB agar plates containing ampicillin and incubated overnight in an inverted position. The following day, a single colony was selected and cultured in shaking conditions. Plasmid DNA was subsequently extracted, and a double digestion was performed using BamHI and EcoRI (Takara, Beijing, China) restriction enzymes. The results of the digestion for the *PLIN2* gene were confirmed through agarose gel electrophoresis.

#### 4.8.3. Analysis of PLIN2 Gene Overexpression and Knockdown Effects

To identify the most effective *PLIN2* interference segment for DPCs, three distinct PLIN2 interference fragments were individually transfected into DPCs. Healthy DPCs derived from Jiangnan cashmere goats were seeded into six-well plates at a density of approximately 3 × 10^5^ cells per well and cultured in a CO_2_ incubator (Thermo Fisher Scientific, model: 371). Upon reaching 30–50% confluency, transfection was conducted. Prior to transfection, each well was refreshed with 2 mL of fresh serum-containing medium devoid of antibiotics. According to the experimental design, DPCs were divided into five groups: the negative control group (NC, empty vector), overexpression control group (oe-NC), *PLIN2* overexpression group (oe-PLIN2), interference control group (si-NC), and *PLIN2* interference group (si-PLIN2), with three replicates per group. Following transfection, cells were cultured for an additional 6 h, after which the medium was replaced with DMEM/F12 supplemented with 10% fetal bovine serum (FBS). Cells were collected 48 h post-transfection for gene expression analysis using a qRT-PCR machine (ABI, model: 7300). Total RNA was extracted using Trizol reagent, and complementary DNA (cDNA) was synthesized utilizing the Hifair^®^ II 1st Strand cDNA Synthesis SuperMix for qPCR kit (Yeasen Biotechnology, Shanghai, China). Target gene expression was quantified using a qPCR SYBR Green kit (Yeasen Biotechnology, Shanghai, China). At 72 h post-transfection, protein expression was evaluated by Western blot analysis. Total protein was extracted using a total protein extraction kit (Solarbio, Beijing, China), and protein concentration was determined with a bicinchoninic acid (BCA) protein concentration assay kit (Solarbio, Beijing, China). The relative expression levels of the RNA were determined with the 2^−ΔΔCT^ method based on the cycle threshold (Ct) values. The primers are detailed in Table 2.

After removing the cells from the incubator, intracellular proteins were extracted according to standard experimental procedures. Following protein extraction, the samples were subjected to Western blot analysis utilizing the same methodology as described previously.

#### 4.8.4. Assessment of PLIN2 Gene Effects on DPC Proliferation, Apoptosis, and Cell Cycle

DPCs in optimal condition were seeded at a density of 5 × 10^3^ cells per well in a 96-well plate and transfected according to five experimental groups, with three replicates for each group. At six hours post-transfection, fresh medium was added to facilitate continued culture. After an additional 12 h, 10 µL of CCK-8 (Servicebio, Wuhan, China) reagent (Apexbio, Houston, TX, USA) was added to each well. Following a 2 h incubation, optical density (OD) values at 450 nm were measured using a microplate reader (Molecular Devices, San Jose, CA, USA, model: SpectraMax M5/M5e) to evaluate cell proliferation.

For apoptosis analysis, the transfected cells were collected, washed twice with PBS (Solarbio, Beijing, China), and centrifuged at 500× *g* for 5 min at 4 °C. The cells were then resuspended in pre-cooled 1× Binding Buffer at a concentration of 1–5 × 10^6^ cells/mL. Subsequently, 100 µL of the cell suspension, combined with 5 µL of Annexin V-FITC/PI apoptosis detection reagent (Apexbio, Houston, TX, USA) and 5 µL of PI staining solution (Solarbio, Beijing, China) was incubated in the dark for 8–10 min. Following this, 400 µL of pre-cooled 1× Binding Buffer was added, and apoptosis was analyzed within 1 h using a flow cytometer (BD Biosciences, Franklin Lakes, NJ, USA, model: BD C6).

To assess the cell cycle, the supernatant was discarded, and the cells were washed with 1 mL of PBS before being digested with 1 mL of 0.25% trypsin (Solarbio, Beijing, China). Once the cells were rounded, digestion was halted by the addition of culture medium, and the cells were resuspended and transferred to centrifuge tubes for a 5 min spin at 1500 rpm. The cells were then resuspended in 1 mL of 75% ethanol that had been pre-cooled to −20 °C and fixed overnight at 4 °C. Following washing with PBS and centrifugation, the cells were resuspended in 100 µL of PBS, and then 2 µL of Recombinant RNase A (Solarbio, Beijing, China) was introduced to remove RNA. The cells were stained in the dark with 100 µL of PI staining solution for 10 min. Cell cycle analysis was performed using a flow cytometer.

### 4.9. Statistical Analysis

Statistical analyses among experimental groups were performed using *t*-tests. All data are presented as mean ± standard error (SE) for comparative purposes. Distinct lowercase letters denote statistically significant differences (*p* < 0.05), whereas identical lowercase letters indicate no significant differences (*p* > 0.05).

## 5. Conclusions

This study conducted proteomic analyses of skin tissues from Jiangnan cashmere goats at three key stages of secondary hair follicle development, identifying multiple proteins associated with this process. Notably, PLIN2 was primarily enriched in the PPAR signaling pathway, highlighting its potential role in regulating lipid metabolism and adipocyte function, both of which are closely linked to hair follicle growth.

Our findings suggest that PLIN2 may influence the hair follicle growth cycle and cashmere fiber production by modulating the proliferation and apoptosis of DPCs. The in-depth investigation of PLIN2 provides critical scientific insights into the molecular mechanisms governing secondary hair follicle development in Jiangnan cashmere goats. Furthermore, this study establishes a theoretical foundation for improving cashmere fiber quality and yield, while also contributing to the optimization of breeding and management strategies.

## Figures and Tables

**Table 1:** Sequence information of *PLIN2* gene interference fragments.

Sequence Name	Primers	Sequence (5′-3′)
si-PLIN2-1	Forward Primer	GCAGAGACCUCUCAUCCUUTT
	Reverse Primer	GUUCAGAAGCCAAGUUAUUAU
si-PLIN2-2	Forward Primer	AGUCUAACAUAAUAACUUGGC
	Reverse Primer	UCAAUCAGGUGAACAGUAGAA
si-PLIN2-3	Forward Primer	CUACUGUUCACCUGAUUGAAU
	Reverse Primer	AAAGCUCGAGUACACCAGCTT
si-NC	Forward Primer	UUCUCCGAACGUGUCACGUTT
	Reverse Primer	ACGUGACACGUUCGGAGAATT

**Table 2:** Quantitative primer information of *PLIN2* gene.

Gene Name	Primers	Sequence (5′-3′)
*GAPDH*	Forward Primer	5′-AGGTCGGTGTGAACGGATTTG-3′
	Reverse Primer	5′-GGGGTCGTTGATGGCAACA-3′
*PLIN2*	Forward Primer	5′-GACCTTGTGTCCTCCGCTTAT-3′
	Reverse Primer	5′-CAACCGCAATTTGTGGCTC-3′

## Data Availability

The original contributions presented in this study are included in the article/Supplementary Materials. Further inquiries can be directed to the corresponding author.

## Supplementary Materials

The following supporting information can be downloaded at: https://www.mdpi.com/article/10.3390/ijms26062710/s1.

## References

[1] Qin C., Du J., Li B., Jia Y., Mai’erhaba A., Ayinuran A. (2023). Investigation on the Development of Cashmere Goat Industry in Southern Xinjiang of Aksu Region. Appl. Eng. Technol..

[2] Yang F., Li R., Zhao C., Che T., Guo J., Xie Y., Wang Z., Li J., Liu Z. (2022). Single-cell sequencing reveals the new existence form of dermal papilla cells in the hair follicle regeneration of cashmere goats. J. Genom..

[3] Yin R., Wang Y., Wang Z., Zhu Y., Cong Y., Wang W., Deng L., Liu H., Guo D., Bai W. (2019). Discovery and molecular analysis of conserved circRNAs from cashmere goat reveal their integrated regulatory network and potential roles in secondary hair follicle. Electron. J. Biotechnol..

[4] Parry A.L., Norton B.W., Restall B.J. (1992). Skin follicle development in the Australian cashmere goat. Aust. J. Agric. Res.

[5] Liu Y., Wang L., Li X., Han W., Yang K., Wang H., Zhang Y., Su R., Liu Z., Wang R. (2018). High-throughput sequencing of hair follicle development-related micrornas in cashmere goat at various fetal periods. Saudi J. Biol. Sci..

[6] Duan C., Zhang L., Gao K., Guo Y., Liu Y., Zhang Y. (2022). Cashmere production, skin characteristics, and mutated genes in crimped cashmere fibre goats. Animal.

[7] Gao Y., Duo L., Zhe X., Hao L., Song W., Gao L., Cai J., Liu D. (2023). Developmental mapping of hair follicles in the embryonic stages of cashmere goats using proteomic and metabolomic construction. Animals.

[8] Fan Y., Rb W., Qiao X., Yj Z., Wang R., Su R., Wu J., Dong Y., Li J. (2015). Hair follicle transcriptome profiles during the transition from anagen to catagen in Cashmere goat (*Capra hircus*). Genet. Mol. Res..

[9] Su R., Gong G., Zhang L., Yan X., Wang F., Zhang L., Qiao X., Li X., Li J. (2020). Screening the key genes of hair follicle growth cycle in Inner Mongolian Cashmere goat based on RNA sequencing. Arch. Anim. Breed..

[10] Bai W.L., Zhao S., Wang Z., Zhu Y., Dang Y.L., Cong Y., Xue H.L., Wang W., Deng L., Guo D. (2018). LncRNAs in Secondary Hair Follicle of Cashmere Goat: Identification, Expression, and Their Regulatory Network in Wnt Signaling Pathway. Anim. Biotechnol..

[11] Müller-Röver S., Handjiski B., Veen C.V., Eichmüller S.B., Foitzik K., McKay I., Stenn K.S., Paus R. (2001). A comprehensive guide for the accurate classification of murine hair follicles in distinct hair cycle stages. J. Investig. Dermatol..

[12] Peus D., Pittelkow M.R. (1996). Growth factors in hair organ development and the hair growth cycle. Dermatol. Clin..

[13] Mifude C., Kaseda K. (2015). PDGF-AA-induced filamentous mitochondria benefit dermal papilla cells in cellular migration. Int. J. Cosmet. Sci..

[14] Zhou N., Fan W., Li M. (2009). Angiogenin is expressed in human dermal papilla cells and stimulates hair growth. Arch. Dermatol. Res..

[15] Kloren W.R.L., Norton B.W., Waters M.J. (1993). Fleece growth in Australian cashmere goats. III. The seasonal patterns of cashmere and hair growth, and association with growth hormone, prolactin and thyroxine in blood. Aust. J. Agric. Res..

[16] Wu J.H., Zhang Y.J., Zhang J.X., Chang Z.L., Li J.Q., Yan Z.W., Zhang W.G. (2012). Hoxc13/β-catenin correlation with hair follicle activity in cashmere goat. J. Integr. Agric..

[17] Geng R., Yuan C., Chen Y. (2013). Exploring differentially expressed genes by RNA-Seq in cashmere goat (*Capra hircus*) skin during hair follicle development and cycling. PLoS ONE.

[18] Liu N., Li H., Liu K., Yu J., Cheng M., De W., Liu J., Shi S., He Y., Zhao J. (2014). Differential expression of genes and proteins associated with wool follicle cycling. Mol. Biol. Rep..

[19] Gao G.L., Zhang Z.Y., Zhang Z.W., Wang W.Z., Nai N.R., Du D.C., Wang W.L., Liu L.Z., Li L.J. (2014). Analysis of Protein Expression Profile of Hair Follicle Cycle of Inner Mongolia Cashmere Goat (*Capra cashmere*). J. Agric. Biotechnol..

[20] Yu M.R., Liu Z.H., Zhang Y.J., Zhao M., Nai R.L., Zheng Z.Q., Li J.Q. (2016). Protein mass spectrometry in the Inner Mongolia White Cashmere Goat. Anim. Husb. Vet. Med..

[21] Yang F. (2020). Mechanisms of Follicular Cell Action in Inner Mongolia Cashmere Goats. Ph.D. Thesis.

[22] Ansari-Renani H.R., Ebadi Z., Moradi S., Baghershah H.R., Ansari-Renani M.Y., Ameli S.H. (2011). Determination of hair follicle characteristics, density and activity of Iranian cashmere goat breeds. Small Rumin. Res..

[23] Kumamoto T., Shalhevet D., Matsue H., Mummert M.E., Ward B.R., Jester J.V., Takashima A. (2003). Hair follicles serve as local reservoirs of skin mast cell precursors. Blood.

[24] Wang H., Yang L., Yu X., He J., Fan L., Dong Y., Dong C., Liu T. (2012). Immunolocalization of β-catenin and Lef-1 during postnatal hair follicle development in mice. Acta Histochem..

[25] Wu C., Li J., Xu X., Xu Q., Qin C., Liu G., Wei C., Zhang G., Tian K., Fu X. (2022). Effect of the FA2H Gene on cashmere fineness of Jiangnan cashmere goats based on transcriptome sequencing. BMC Genom..

[26] Wu J., Li Y., Gong H., Wu D., Li C., Liu B., Ding L. (2020). Molecular Basis of Maintaining Circannual Rhythm in the Skin of Cashmere Goat. bioRxiv.

[27] Wu C., Qin C., Fu X., Huang X., Tian K. (2022). Integrated analysis of lncRNAs and mRNAs by RNA-Seq in secondary hair follicle development and cycling (anagen, catagen and telogen) of Jiangnan cashmere goat (*Capra hircus*). BMC Vet. Res..

[28] Zouboulis C.C., Nogueira da Costa A., Makrantonaki E., Hou X., Almansouri D., Dudley J.T., Edwards H., Readhead B., Balthasar O., Jemec G.B. (2020). Alterations in innate immunity and epithelial cell differentiation are the molecular pillars of hidradenitis suppurativa. J. Eur. Acad. Dermatol. Venereol..

[29] Wu C. (2022). Screening of Cashmere Trait Related Genes and Functional Verification of ELOVL3 and FA2H Genes in Jiangnan Cashmere Goats. Ph.D. Thesis.

[30] Zhang Y., Huang J., Fu D., Liu Z., Wang H., Wang J., Qu Q., Li K., Fan Z., Hu Z. (2021). Transcriptome analysis reveals an inhibitory effect of Dihydrotestosterone-treated 2D-and 3D-cultured dermal papilla cells on hair follicle growth. Front. Cell Dev. Biol..

[31] Pan X., Hobbs R.P., Coulombe P.A. (2013). The expanding significance of keratin intermediate filaments in normal and diseased epithelia. Curr. Opin. Cell Biol..

[32] Ohnemus U., Uenalan M., Inzunza J., Gustafsson J.A., Paus R. (2006). The hair follicle as an estrogen target and source. Endocr. Rev..

[33] Hearle JW S. (2000). A critical review of the structural mechanics of wool and hair fibres. Int. J. Biol. Macromol..

[34] Akiba H., Ikeuchi E., Ganbat J., Fujikawa H., Arai-Kusano O., Iwanari H., Nakakido M., Hamakubo T., Shimomura Y., Tsumoto K. (2018). Structural behavior of keratin-associated protein 8.1 in human hair as revealed by a monoclonal antibody. J. Struct. Biol..

[35] Langbein L., Rogers M.A., Winter H., Praetzel S., Beckhaus U., Rackwitz H.R., Schweizer J. (1999). The catalog of human hair keratins. I. Expression of the nine type I members in the hair follicle. J. Biol. Chem..

[36] Yamamoto M., Sakamoto Y., Honda Y., Koike K., Nakamura H., Matsumoto T., Ando S. (2021). De novo filament formation by human hair keratins K85 and K35 follows a filament development pattern distinct from cytokeratin filament networks. FEBS Open Bio.

[37] Plowman J.E., Harland D.P., Richena M., Thomas A., Hefer C.A., van Koten C., Scobie D.R., Grosvenor A.J. (2022). Wool fiber curvature is correlated with abundance of K38 and specific keratin-associated proteins. Proteins.

[38] Jin M., Wang J., Chu M.X., Piao J., Piao J.A., Zhao F.Q. (2016). The study on biological function of keratin 26, a novel member of Liaoning cashmere goat keratin gene family. PLoS ONE.

[39] Duchstein P., Clark T., Zahn D. (2015). Atomistic modeling of a KRT35/KRT85 keratin dimer: Folding in aqueous solution and unfolding under tensile load. Phys. Chem. Chem. Phys..

[40] Hui T., Zheng Y., Yue C., Wang Y., Bai Z., Sun J., Cai W., Zhang X., Bai W.L., Wang Z. (2021). Screening of cashmere fineness-related genes and their ceRNA network construction in cashmere goats. Sci. Rep..

[41] Houschyar K.S., Borrelli M.R., Tapking C., Popp D., Puladi B., Ooms M., Chelliah M.P., Rein S., Pförringer D., Thor D. (2020). Molecular mechanisms of hair growth and regeneration: Current understanding and novel paradigms. Dermatology.

[42] Ohnemus U.G., Uenalan M., Conrad F., Handjiski B., Mecklenburg L., Nakamura M., Inzunza J., Gustafsson J., Paus R. (2005). Hair cycle control by estrogens: Catagen induction via estrogen receptor (ER)-α is checked by ERβ signaling. Endocrinology.

[43] Moraleva A.A., Deryabin A., Rubtsov Y.P., Rubtsova M.P., Dontsova O.A. (2022). Eukaryotic ribosome biogenesis: The 40S subunit. Acta Naturae.

[44] Zhang C., Qin Q., Liu Z., Wang Y., Lan M., Zhao D., Zhang J., Wang Z., Li J., Liu Z. (2024). Combining multiomics to analyze the molecular mechanism of hair follicle cycle change in cashmere goats from Inner Mongolia. Front. Vet. Sci..

[45] Festa E., Fretz J.A., Berry R., Schmidt B., Rodeheffer M.S., Horowitz M.C., Horsley V. (2011). Adipocyte lineage cells contribute to the skin stem cell niche to drive hair cycling. Cell.

[46] Nepal S., Venkataram A., Mysore V. (2021). The Role of Adipose Tissue in Hair Regeneration: A Potential Tool for Management?. J. Cutan. Aesthet. Surg..

[47] Bai T., Liang B., Zhao Y., Han J., Pu Y., Wang C., Ma Y., Jiang L. (2021). Transcriptome analysis reveals candidate genes regulating the skin and hair diversity of Xinji fine-wool sheep and Tan sheep. Agriculture.

[48] Icre G., Wahli W., Michalik L. (2006). Functions of the peroxisome proliferator-activated receptor (PPAR) alpha and beta in skin homeostasis, epithelial repair, and morphogenesis. J. Investig. Dermatol. Symp. Proc..

[49] Zhou X., Bao P., Zhang X., Guo X., Liang C., Chu M., Wu X., Yan P. (2022). Genome-wide detection of RNA editing events during the hair follicles cycle of Tianzhu white yak. BMC Genom..

[50] Yin N., Zhang J.J. (2021). Autotaxin-LPA Axis in Obesity and Obesity-related Diseases. Prog. Biochem.Biophys..

[51] Najt C.P., Devarajan M., Mashek D.G., Devarajan M., Mashek D.G. (2022). Perilipins at a glance. J. Cell. Sci..

[52] Singh R., Kaushik S., Wang Y., Xiang Y., Novak I., Komatsu M., Tanaka K., Cuervo A.M., Czaja M.J. (2009). Autophagy regulates lipid metabolism. Nature.

[53] Tsai T., Chen E., Li L., Saha P.K., Lee H., Huang L., Shelness G.S., Chan L., Chang B.H. (2017). The constitutive lipid droplet protein PLIN2 regulates autophagy in liver. Autophagy.

[54] Imamura M., Inoguchi T., Ikuyama S., Taniguchi S., Kobayashi K., Nakashima N., Nawata H. (2002). ADRP stimulates lipid accumulation and lipid droplet formation in murine fibroblasts. Am. J. Physiol. Endocrinol. Metab..

[55] Horton P., Park K.J., Obayashi T., Fujita N., Harada H., Adams-Collier C.J., Nakai K. (2007). WoLF PSORT: Protein localization predictor. Nucleic Acids Res..

[56] Robinson M.D., McCarthy D.J., Smyth G.K. (2010). edgeR: A Bioconductor package for differential expression analysis of digital gene expression data. Bioinformatics.

[57] Kanehisa M., Goto S. (2000). KEGG: Kyoto encyclopedia of genes and genomes. Nucleic Acids Res..

[58] Szklarczyk D., Gable A.L., Lyon D., Junge A., Wyder S., Huerta-Cepas J., Simonovic M., Doncheva N.T., Morris J.H., Bork P. (2019). STRING v11: Protein–protein association networks with increased coverage, supporting functional discovery in genome-wide experimental datasets. Nucleic Acids Res..

