# Supplementary figures and images for: Proteomics Reveals the Role of PLIN2 in Regulating the Secondary Hair Follicle Cycle in Cashmere Goats
Source: Int J Mol Sci. 2025 Mar 18;26(6):2710. doi: 10.3390/ijms26062710 (PMC11942475; doi:10.3390/ijms26062710)

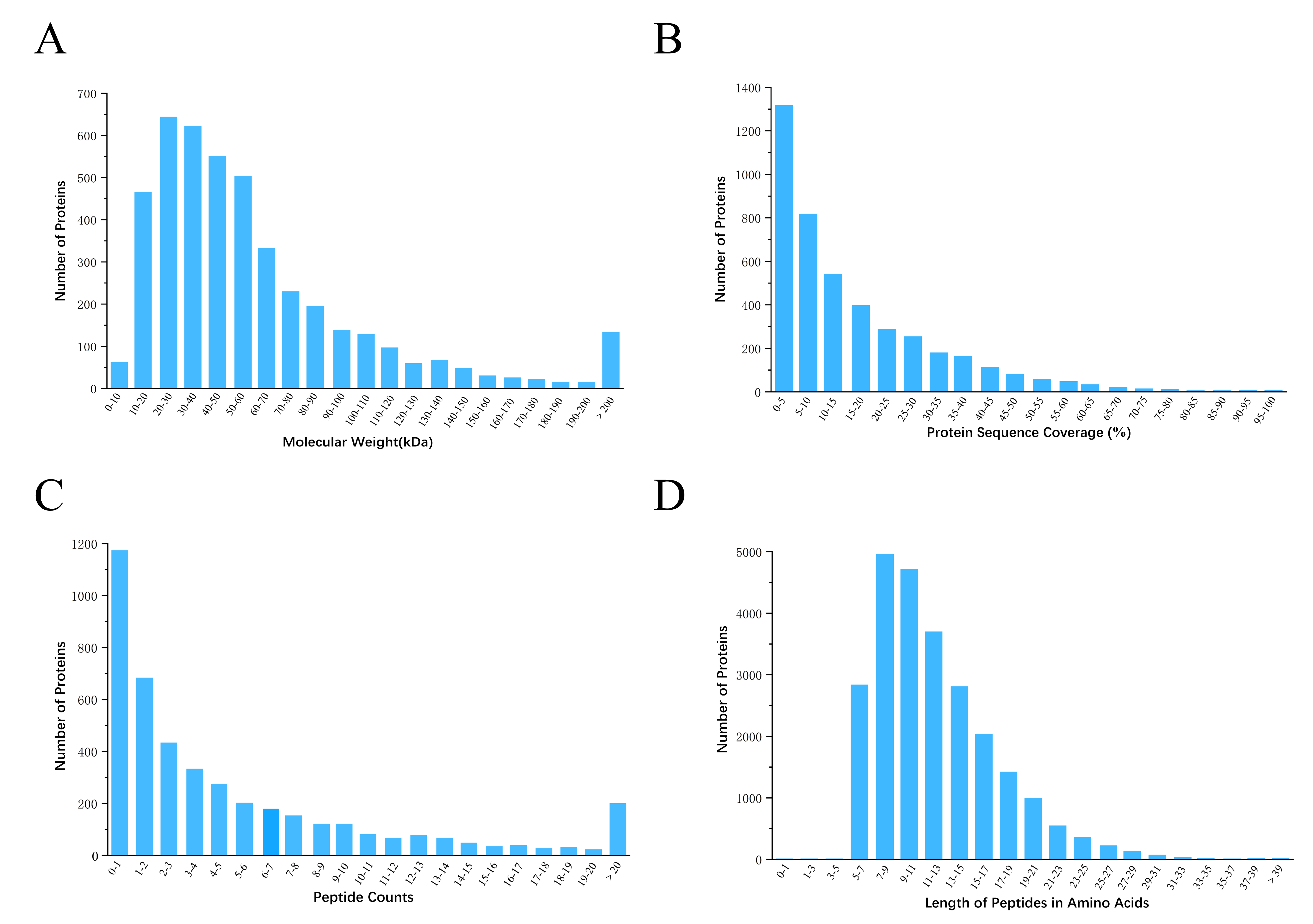

Supplement: Supplementary file 1 [file ijms-26-02710-s001.zip › ijms-3489948-supplementary/Supplementary File/Supplementary Figures/Figure S1 Protein Functional Analysis in Skin Tissue of Jiangnan cashmere goats/Figure S1 Protein Functional Analysis in Skin Tissue of Jiangnan cashmere goats.png]

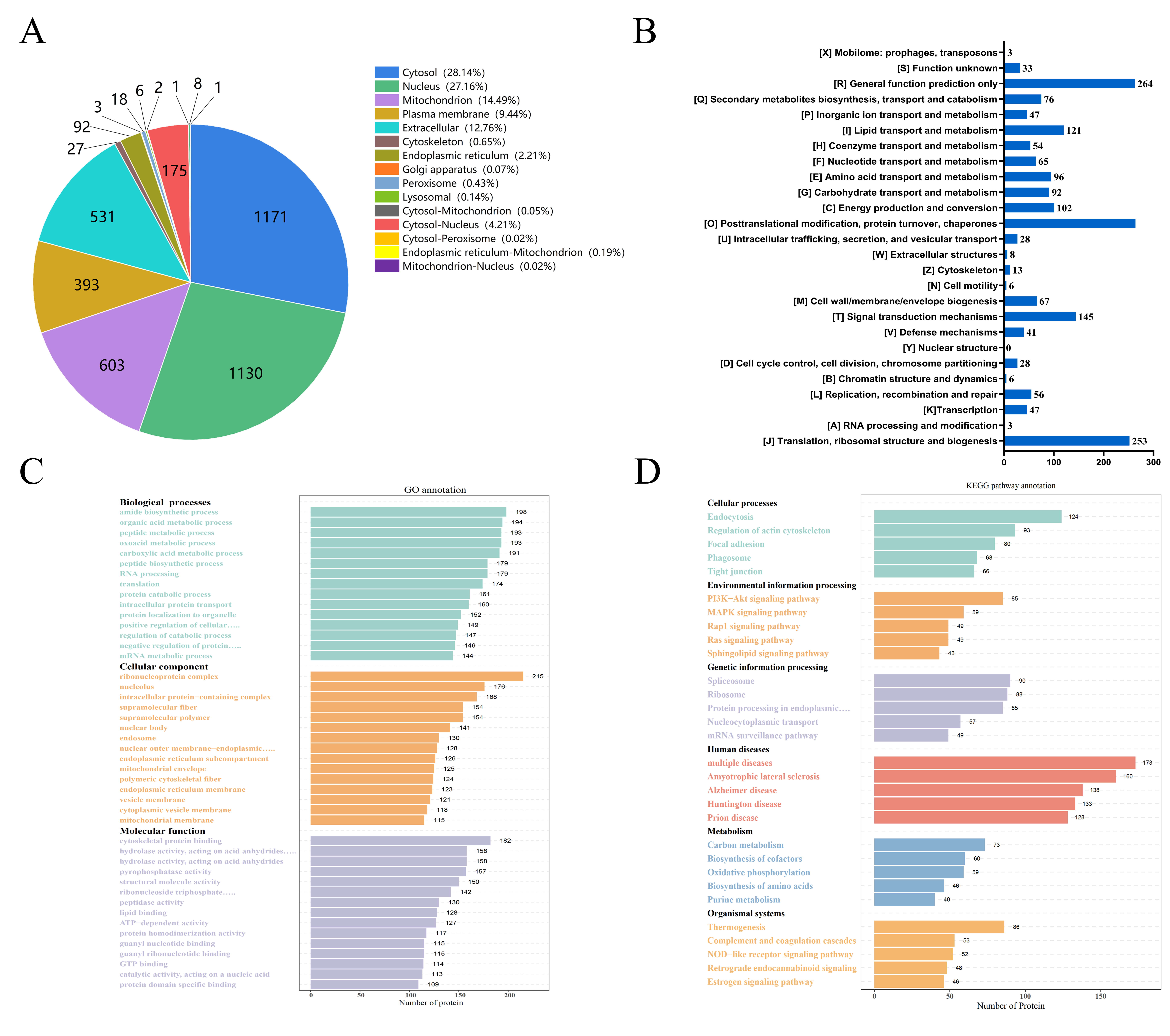

Supplement: Supplementary file 1 [file ijms-26-02710-s001.zip › ijms-3489948-supplementary/Supplementary File/Supplementary Figures/Figure S2 Molecular mass distribution, sequence coverage and peptide composition of proteins/Figure S2 Molecular mass distribution, sequence coverage and peptide composition of proteins.png]

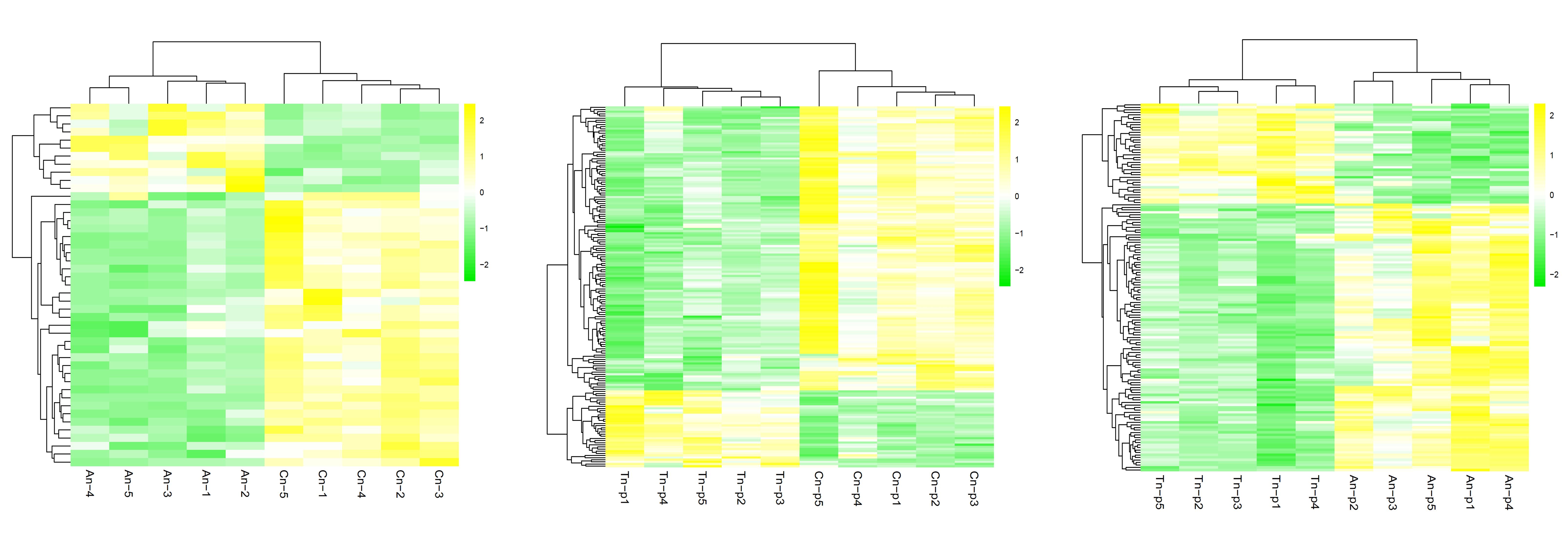

Supplement: Supplementary file 1 [file ijms-26-02710-s001.zip › ijms-3489948-supplementary/Supplementary File/Supplementary Figures/Figure S4 Heat map of DEPs/Figure S4 Heat map of DEPs.png]

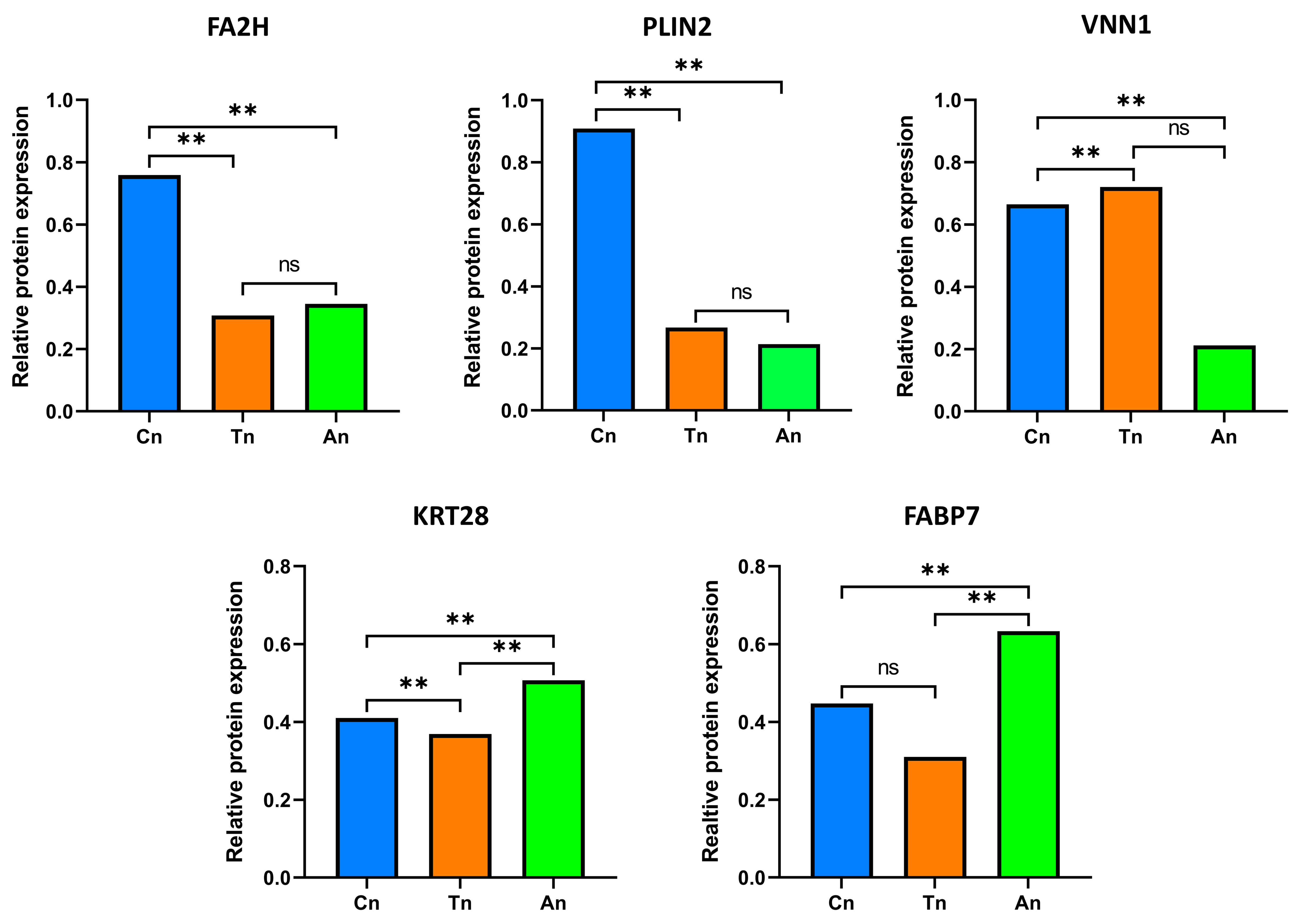

Supplement: Supplementary file 1 [file ijms-26-02710-s001.zip › ijms-3489948-supplementary/Supplementary File/Supplementary Figures/Figure S5 Histogram of quantitative analyses of the proteins FA2H, PLIN2, VNN1, KRT28, and FABP/Figure S5 Histogram of quantitative analyses of the proteins FA2H, PLIN2, VNN1, KRT28, and FABP.png]
